# Virtual Reality as a Potential Tool to Face Frailty Challenges

**DOI:** 10.3389/fpsyg.2017.01541

**Published:** 2017-09-05

**Authors:** Silvia Serino, Serena Barello, Francesca Miraglia, Stefano Triberti, Claudia Repetto

**Affiliations:** ^1^Department of Psychology, Catholic University of the Sacred Heart Milan, Italy; ^2^Applied Technology for Neuropsychology Lab, Istituto Auxologico Italiano Milan, Italy; ^3^Department of Geriatrics, Neuroscience and Orthopedics, Institute of Neurology, Catholic University of the Sacred Heart Rome, Italy; ^4^Brain Connectivity Laboratory, IRCCS San Raffaele Pisana Rome, Italy

**Keywords:** virtual reality, frailty, rehabilitation, executive functions, gait, aging in place, patient engagement

The aging population and the corresponding increase in age-related diseases present scientific community and public health authorities with imminent challenges. One of these challenges deals with a deeper understanding of functional status of elderly in order to prevent and/or delay the onset of late-life disability (Rodríguez-Artalejo and Rodríguez-Mañas, 2014). The syndrome of “frailty” has been recently introduced in literature to specifically characterize the health of older individuals who deserve special attention because of their increased vulnerability to adverse health outcomes (Afilalo et al., 2010). Although there is not a unique definition of frailty (Morley et al., 2013), the majority of studies refers to the five operational criteria (Fried et al., 2001): decreased gait speed, reduced grip strength, prolonged and unmotivated exhaustion, low physical activity, unintended weight loss. The problem of different definitions leads also to a large variation in reported prevalence rates, which range approximately from 5 to 60% (Collard et al., 2012). However, this multifaceted decline in different physiological systems make frail older individuals progressively more exposed to stressors (Clegg et al., 2013), making urgent the need for better care interventions.

In parallel, some authors suggested to introduce also the phenotype of “cognitive frailty” to refer to older individuals who manifest a concurrent weakness in both physical and cognitive domains (Kelaiditi et al., 2013). Other than the presence of physical frailty, Kelaiditi and colleagues (Kelaiditi et al., 2013) proposed that the key criteria of cognitive frailty is the presence of mild cognitive impairment, in the absence of dementia. A very recent large study (Delrieu et al., 2016), involving 1.617 participants, clarified that cognitive frailty individuals showed a specific weakness in executive domain (i.e., a wide range of high-level cognitive abilities including problem-solving, planning, monitoring). These findings were in line with studies suggesting a crucial link between an early cognitive decline in frontal areas and gait deficits (Montero-Odasso et al., 2011), since they share common brain networks.

Different treatment approaches have been investigated in clinical trials for reducing the functional decline of frail individuals: exercise interventions (Forster et al., 2009), nutritional programs (Fiatarone et al., 1994), and integrated approaches (Looman et al., 2016). Three main issues emerged so far: the need for a more precise identification of markers of frailty; a call for innovative therapeutic strategies; the importance to develop personalized and integrated care models and intervention approaches aimed at improving independence, preferably delivered in their home setting (de Vos et al., 2012). In this context, we suggest that Virtual Reality can be an innovative tool potentially able to address the aforementioned issues.

## Virtual reality for frail patients: how and why

Virtual Reality (VR) is a combination of technological devices that allows users to navigate into and interact with tridimensional computer-created environments, having the subjective sensation to be there (Sanchez-Vives and Slater, 2005; Triberti and Riva, 2015). In the last decades, VR has been extensively used in neuroscience, and recent studies investigated the role of VR on brain modulation via neuroimaging methods. These studies aimed to characterize brain activity in virtual environments in order to understand the neurophysiologic correlates of the virtual navigation (Pacheco et al., 2017). Neuroimaging evidence suggested that medial temporal lobe structures, the hippocampus in particular, as well as parietal and frontal regions have been implicated in spatial navigation in humans (Iaria et al., 2008). Furthermore, it was observed an elevated theta and gamma power associated with VR navigation and increased theta coherence between right parietal and temporal regions (Cornwell et al., 2008). Theta and gamma oscillatory activity and cortico-hippocampal communication are part of a brain mechanism involved in the information transfer of different spatial representations required for successful navigation (White et al., 2012); parietal lobe is involved in numerous aspects of visuo-spatial cognition; theta and gamma activity in this region likely reflects the mechanism by which medial temporal and parietal brain regions communicate during navigation (Moser et al., 2008).

Therefore, experimental evidence underlined that during navigation in a virtual environment the brain activity is modulated (Weidemann et al., 2009), and paved the way to consider VR a potential tool for diagnosis and rehabilitation of motor and cognitive deficits.

Along this line of reasoning, in the following paragraphs we want to clarify how VR can provide interesting opportunities in order to deal with the challenges prompted by frailty: early symptoms identification, motor and cognitive rehabilitation, home-setting interventions.

As far as motor symptoms are concerned, an increasing number of studies have developed novel paradigms to deeply model the gait characteristics (e.g., Martens et al., 2017), especially for those ones difficult to analyze in clinical setting (i.e., intermittent gait freezing in Parkinson's Disease—PD, or—more related to frailty—a decrease in gait speed). For instance, Shine and co-workers developed a VR-based environment for investigating gait features (Shine et al., 2013). In this paradigm, the participants, seated in front of a computer screen, were asked to navigate in a realistic virtual environment (i.e., a corridor) via footpedals. In addition to the “simple command” (such as “WALK” or “STOP” that appeared on the screen), they were trained to more complex stimuli, entailing executive functions: the congruent color-word (“BLUE” written in blue that means “WALK”), or the incongruent color-word (“RED” written in green that means “STOP”). Shine and co-workers (Shine et al., 2013) found that PD patients with freezing of gait had a large frequency of motor arrests in comparison with “non-freezers” PD patients on this task, making it suitable for modeling the gait behavior. However, this dual-task paradigm implemented in VR appears also particularly useful for the evaluation of motor aspects of gait speed and related cognitive deficits. As far as cognitive symptoms are concerned, traditional paper-and-pencil tests are not reliable to capture the “complexity” of executive functioning emerging in real-life situations (Shallice and Burgess, 1991; Goldstein, 1996). One attempt to overcome this issue is the development of tests evaluating the executive functioning in real-life scenarios, such as the Multiple Errands Test (a shopping task in a supermarket, Shallice and Burgess, 1991) or the Executive Function Performance Test (simple cooking, telephone use, and medication management, Baum et al., 2008). Given the difficulties in reproducing these tests in real life situations (i.e., time consuming, high economic costs, safety of the patients, poor controllability of experimental conditions), VR technology has been increasingly used for the assessment of executive functions. Indeed, VR permits to develop scenarios reproducing daily-life situations, allowing a secure and ecologically valid assessment of executive functions (Parsons, 2011). For example, Nir-Hadad and co-workers (Nir-Hadad et al., 2017) recently developed and tested in a sample of 19 post-stroke patients a virtual version of the original Four Item Shopping Task, which requires budget management as a functional test of executive functioning. Also the virtual version of the Multiple Errands Test has been developed and tested in different clinical populations (Raspelli et al., 2012; Cipresso et al., 2014).

Second, VR could be a promising tool to enhance neuroplasticity in neurorehabilitation (Ng et al., 2013). The concept underlying VR-based therapy as a treatment for motor and cognitive dysfunction is to improve neuroplasticity of the brain by engaging users in multisensory training. VR-based intervention effectiveness was demonstrated in several chronic stroke patients (Lloréns et al., 2015), in vestibular (Alahmari et al., 2014), in sensori-motor (Fluet and Deutsch, 2013) and cognitive rehabilitation of neurological patients (Slobounov et al., 2015).

A recent systematic review found that VR-based trainings were more effective than conventional therapies in enhancing balance and gait ability in post-stroke patients (de Rooij et al., 2016). The advantages offered by VR over conventional approaches were multiple: within virtual environments, it is possible to develop repetitive and personalized motor training that are enriched by different feedbacks (proprioceptive, visual, auditory) able to maximize motor learning. In particular, the use of VR in combination with haptic devices (i.e., robotic systems able of give users tactile and force feedbacks when interacting with virtual objects) can enhance the environment realism (Hoffman et al., 1998), thus improving the efficacy of a rehabilitation program (Teruel et al., 2015). Moreover, VR-based stimulation can provide frail individuals with engaging and enriching environments, helping them to repeat the exercises harder and longer, thus exploiting the principle of motor learning (Kitago and Krakauer, 2013). Furthermore, although VR is currently in the developmental phase in terms of treatment of frailty (Mugueta-Aguinaga and Garcia-Zapirain, 2017), VR-based rehabilitation protocols have been already tested for training executive functions in other clinical populations (Faria et al., 2016). It is worthy to underline that cognitive training might be particularly demanding for elderly, especially in case of cognitive impairment. As previously explained, VR offers the chance to set-up cognitive exercises within meaningful environments (Riva et al., 2006). Moreover, in virtual environments it is possible to reproduce real-life situation in a safer and more controlled setting: the ecological validity is an important feature in neuropsychological assessment and remediation, even more so for the executive functions trainings.

Finally, as the old and frail population continues to grow, a great deal of attention has been dedicated to find organizational solutions aimed at promoting *aging-in-place* policies, in order to facilitate individuals in living independently in one's own home as long as possible (Stones and Gullifer, 2016). Indeed, *aging-in-place* is recognized as a crucial strategy to improve the quality of life of elderly citizens as well as the sustainability of social and welfare systems. Early evidence is supportive of the advantages of structured program to enable *aging-in-place* as it enhances patient engagement in their own medical and rehabilitation processes (Kim et al., 2017), which is a crucial predictor of patients' quality of life and medication adherence (Barello and Graffigna, 2015). However, *aging-in-place* requires a reframing of the care models in terms of seamless transitions between hospitals, the welfare system and territory care, along with all other physical and social contexts in an elderly citizens life. However, accomplishing this will require substantial innovation in the incorporation of advanced technologies in the process of care and cure. In this context, VR-based technologies might be a powerful tool to make the *aging-in-place* imperative a concrete reality (Lange et al., 2010). Indeed, this tool might guarantee elderly people to follow the rehabilitation process directly at home. Moreover, VR has the potential to sustain elderly people active engagement in the medical course due to their high level of customization according the patient's unique expectations and care needs (Graffigna et al., 2014). According to these reflections, *aging-in-place* using VR-based technologies may be a promising solution for the upcoming aging society. However, the implementation of these solutions at home should consider also the introduction of some specific systems that allow patients' monitoring (e.g., intelligent systems for teletherapy, Rodríguez et al., 2016, or wearable devices for unobtrusive monitoring, Patel et al., 2012) to make VR-based training as controlled as in clinical settings.

Beside the numerous advantages VR offers for facing frailty challenges, potential limitations should also be taken into account. At a basic research level, it should be acknowledged that there are still few evidences about of VR brain modulation effects in several domains: long-term outcomes, direct comparisons between commercial and customized modules, immersive vs. non-immersive VR, and augmented vs. fully virtual systems. At the applicative level, frail older adults could show low degrees of technology acceptance, due to a general diffidence toward the technological devices or to the discomfort elicited by the specific VR set up proposed. These limitations, thought, should not prevent researchers and clinicians from carrying on projects that test the use of VR with elderly and frail patients; on the contrary, these issues should stimulate to move forward in basic and applicative research in order to better exploit in the future the VR capabilities with frail individuals.

## Author contributions

SS, SB, FM, and CR conceived the work. SS, SB, ST, and FM drafted the paper. CR and ST revised critically the work.

### Conflict of interest statement

The authors declare that the research was conducted in the absence of any commercial or financial relationships that could be construed as a potential conflict of interest.
